# Cement Augmentation of Two-Level Lumbar Corpectomy Cage After Malposition: A Novel Salvage Procedure Technical Note

**DOI:** 10.7759/cureus.29074

**Published:** 2022-09-12

**Authors:** Mousa K Hamad, Jessica Ryvlin, Justin Langro, Aisha S Obeidallah, Jason Marin, Rafael De La Garza Ramos, Saikiran Murthy, Seon-Kyu Lee, Reza Yassari

**Affiliations:** 1 Neurological Surgery, Montefiore Medical Center, New York, USA; 2 Neurointerventional Radiology, Montefiore Medical Center, New York, USA

**Keywords:** neurointerventional radiology, cement augmentation, vertebroplasty, intervertebral cage, lumbar spine, salvage procedure, salvage, vertebral osteomyelitis, corpectomy

## Abstract

Intervertebral cage mispositioning is an uncommon complication of a posterior lumbar corpectomy. Most frequently, cages are placed obliquely, laterally, or protruding. However, there are few reports of implanted cages that fail to contact the adjacent vertebral endplate and thus no descriptions of successful revisions. The objective of this case report is to report a unique case of minimally invasive rescue vertebroplasty with cement augmentation following a lumbar corpectomy that resulted in graft-endplate noncontact in a medically complicated patient

A 60-year-old male with a history of active intravenous (IV) drug use, untreated hepatitis C virus (HCV) infection, and chronic malnourishment presented with low back pain. He had a history of vertebral osteomyelitis managed with intravenous antibiotics, although he was noncompliant with infusions. The diagnosis of L2-L3 discitis-osteomyelitis with intradiscal abscess causing cord compression was made using inpatient lumbar imaging. The initial intervention was accomplished with L2 and L3 vertebral corpectomy with decompression and expandable cage placement as well as a T10-pelvis posterior fixation. Despite the resolution of presenting symptoms, routine postoperative radiographs identified noncontact between the inferior surface of the cage and the superior endplate of the L4 vertebral body. Salvage therapy was pursued via fluoroscopy-guided vertebroplasty with cement augmentation to correct cage malposition. Secondary surgical intervention was successful in bringing the intervertebral cage into contact with the adjacent vertebral body. Lower extremity strength improved, and back pain was resolved. The postoperative motor examination remained unchanged after the rescue procedure. Accurate intraoperative cage placement can be difficult in patients with poor bone quality, especially in the setting of ongoing infection and cachexia. For this reason, routine postoperative imaging is crucial to assessing graft complications. In patients who are poor candidates for revision surgery, we demonstrate that an interventional radiology-based approach may be successful in correcting cage mispositioning and preventing further changes during healing and fusion.

## Introduction

Vertebral corpectomies are commonly utilized for the treatment of osteomyelitis, tumor debulking, and compression fractures. Recently, expandable cages have been more often preferred for placement due to their superiority in deformity correction and decreased risk of subsidence [1,2].

Accurate cage placement can prove challenging during corpectomy, especially in patients with poor bone quality or distorted/collapsed anatomy as a result of infection. In the lumbar spine, especially, proper cage placement can be difficult due to its lordotic nature [3]. The most common complications of corpectomy cage placement include screw loosening and failure, cage subsidence, and cage dislocation [4-8]. Expandable cage subsidence in particular can be seen in nearly half of patients who undergo vertebral body corpectomy in the cervical and lumbar spine [9,10]. This is likely increased in patients who undergo multilevel corpectomy.

Revision of observed complications such as mispositioning has not been well described in the literature. Prevention is better understood, including adjusting the shape of the endcaps and utilizing intraoperative navigation to reduce rates of misplacement [3,11,12]. To the best of our knowledge, there have been no reports of intervertebral cages that fail to contact the adjacent vertebral body and thus no reports of attempted revisions. In this case report, we describe a successful salvage procedure for a malpositioned expandable corpectomy cage in a medically complex patient with progressive osteomyelitis.

## Case presentation

A 60-year-old cachectic male with active intravenous (IV) drug use and prior osteomyelitis was transferred to our emergency department for evaluation of progressing vertebral osteomyelitis and epidural abscess. His chief complaint was worsening lower back pain. Of note, prior history also included untreated hepatitis C virus (HCV), opioid use disorder on methadone with concurrent IV drug use, hypertension, diabetes mellitus, coronary artery disease status post-coronary artery bypass grafting (CABG), and chronic malnutrition. On admission, two peripheral blood cultures confirmed bacteremia with methicillin-sensitive *Staphylococcus aureus* (MSSA). Physical examination showed an isolated decrease in hip flexion strength (3/5) along with pain-limited weakness in ankle plantarflexion and dorsiflexion. Deep tendon reflexes were 2+ and symmetric throughout.

Imaging findings

**Figure 1:**
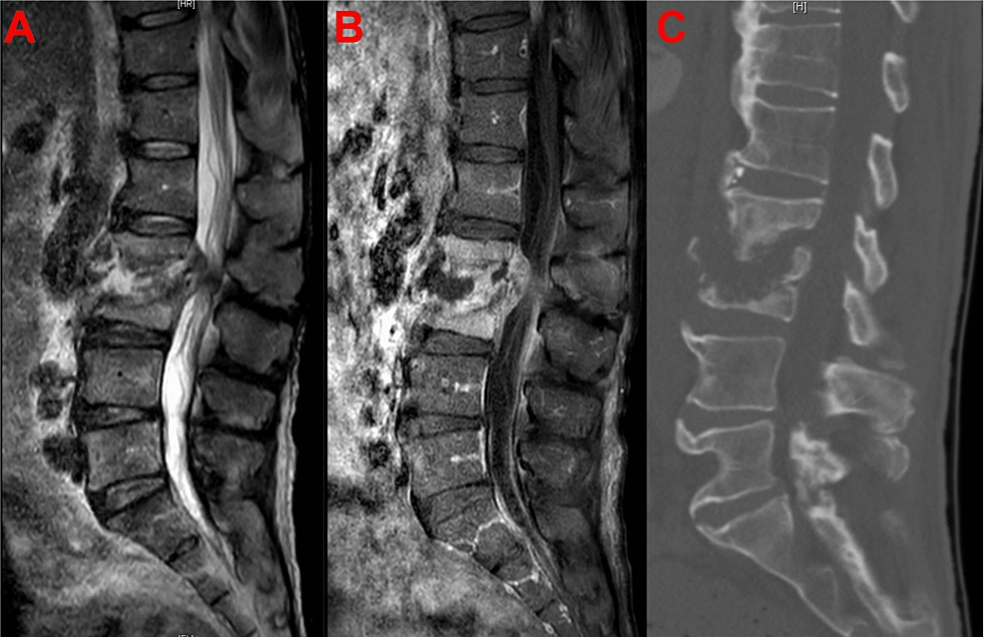
(A) Midsagittal T2-weighted MRI without contrast. (B) Midsagittal T1-weighted MRI with contrast. (C) Midsagittal CT scan without contrast. Imaging prior to initial intervention showing L2 and L3 discitis-osteomyelitis with intradiscal abscess causing cord compression.

Preoperative lumbar CT revealed endplate erosion and partial vertebral body collapse at L2 and L3 with severe spinal canal stenosis. MRI of the same area confirmed the presence of discitis-osteomyelitis at L2 and L3 with intradiscal abscess and posterior epidural phlegmon causing thecal sac compression. Intramuscular abscess formation was present in the bilateral psoas and iliacus muscles at the L2 and L3 levels (Figure 1A-1C).

Initial intervention

**Figure 2:**
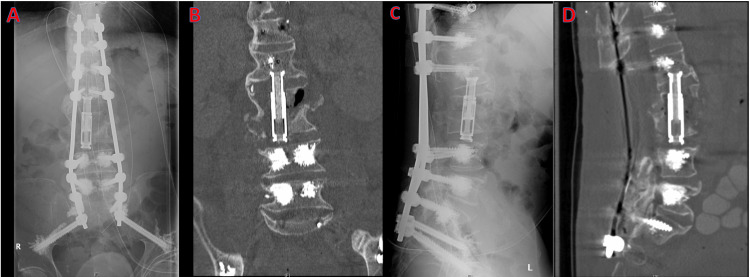
Postoperative films: (A) anteroposterior projection X-ray, (B) coronal CT scan, (C) lateral projection X-ray, and (D) sagittal CT scan. Note that the inferior surface of the graft does not contact the superior L4 endplate and the top of the graft has subsided into the L1 body.

The patient underwent uncomplicated T10-pelvis posterior fusion with cement augmentation and L2-L3 corpectomy with expandable cage placement. Direct visualization intraoperatively demonstrated superior and inferior endplate contact. Manual manipulation of the graft also demonstrated good purchase (Figure 2). Vertebroplasty at T9 was deferred due to the length of surgery and copious blood loss we encountered.

Rescue intervention

**Figure 3:**
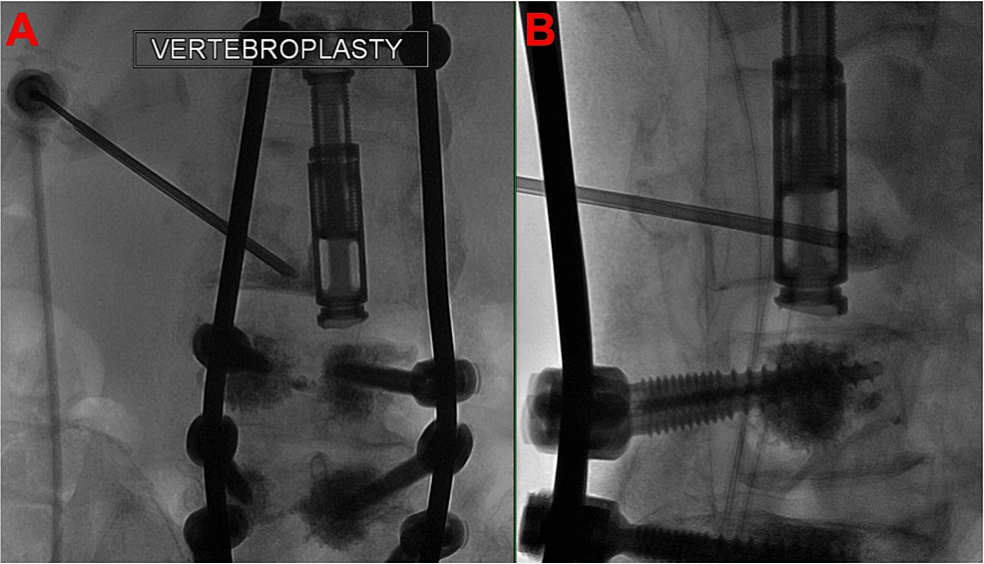
Vertebroplasty needle placement: (A) anteroposterior fluoroscopy and (B) lateral fluoroscopy.

**Figure 4:**
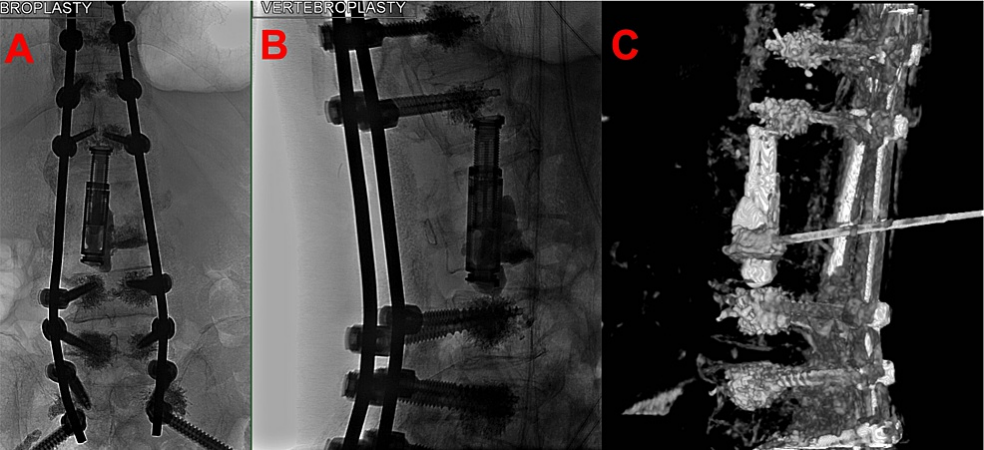
(A) Anteroposterior, (B) lateral, and (C) three-dimensional reconstruction after XperCT, which emphasizes the cement injected inside the graft, anterior lateral and inferiorly now contacting the superior endplate of L4.

A minimally invasive approach was pursued given this patient’s significant comorbidities and patient preference to avoid a second large operation. To correct the floating intervertebral cage, a fluoroscopically guided salvage vertebroplasty with cement augmentation was performed using an extrapedicular approach (Figures 3, 4). The left pedicle of L3 was identified using a far lateral approach, and the needle was targeted immediately lateral to the pedicle to access the L3 vertebral body. Using biplane fluoroscopic guidance to avoid pedicle breach, the needle was advanced to the target vertebral body. Then, a poly(methyl methacrylate) (PMMA) mixture was injected through the pedicular needle under fluoroscopic guidance. A total of 4.5 cc of cement was filled from pedicle to pedicle and vertically to each endplate at the L3 level, which was confirmed on XperCT. Vertebroplasty at T9 was performed to prevent proximal junctional kyphosis.

Results

Following the salvage procedure, the patient reported complete resolution of back pain, unchanged following the initial intervention. The postoperative neurological examination was also unchanged, with stable 5/5 lower extremity strength and no change in sensation. The patient was discharged to a subacute rehabilitation facility for long-term IV antibiotic administration after a total of 40 days spent in the inpatient setting. Following discharge, the patient became lost to follow-up, so long-term reevaluation was unable to be completed.

## Discussion

Cage placement is often challenging in patients with chronic cachexia or those with poor bone quality [13,14]. The commonly documented complications following vertebral corpectomy and intervertebral expandable cage implantation include subsidence, cage migration, and screw failure, which can lead to neurological compromise, spinal instability, and damage to surrounding structures and vasculature. For these complications, cement augmentation can be successfully used to restore normal anatomy [15]. However, reports of postoperative migrating or “floating” cages and recommended treatment courses have not been clearly documented in the literature.

Following postoperative imaging and identification of the cage placement complication, the surgical team had a thorough discussion with the patient regarding corrective options. The possibilities ranged from cage removal and exchange to close follow-up. He opted for a minimally invasive option that did not involve replacement or revision of the implants.

While screw placement was intraoperatively navigated, including CT imaging before and after instrumentation, we question whether an additional CT after deformity correction but before closure would have shown evidence of early subsidence. However, intraoperative radiographs following initial cage expansion did not demonstrate malposition. Ultimately, it is possible that the implant artifact may have interfered with adequate graft and endplate visualization. Another potential source of failure in this patient was the decision to pursue all-posterior surgical intervention, as opposed to a direct lateral corpectomy for rescue. Although a lateral approach may have provided a larger graft and endplate area, thus decreasing the risk of subsidence, we decided on a posterior approach to preserve the psoas given the concurrent psoas abscess.

One of our wise reviewers asked an important question regarding options if there is continued graft subsidence on repeat imaging. Given the cement above and below the graft, we feel that the risk is minimal, but employing a more invasive direct lateral technique to remove the graft (while entailing more risk to the psoas muscle), possibly including L1 in the corpectomy (if the vertebral body loses its structural integrity) and deploying a larger expandable cage with a larger endplate footprint would be necessary.

## Conclusions

Accurate intraoperative expandable cage placement can be difficult in patients with poor bone quality, especially in the setting of ongoing infection and cachexia. In patients who are poor surgical candidates, we demonstrate that a minimally invasive interventional radiology approach may be successfully pursued for correcting cage mispositioning and preventing further changes during healing and fusion. Although revision surgery utilizing an alternative approach was our first choice, thorough risk/benefit analysis and discussion with the patient led to the development of this salvage technique. This case report describes a successful salvage procedure in a medically complex patient that may be used to guide future corrective attempts and highlights the importance of routine postoperative imaging to confirm the adequate placement of grafts in neurosurgical and orthopedic spine procedures.
